# Hybridization and postzygotic isolation promote reinforcement of male mating preferences in a diverse group of fishes with traditional sex roles

**DOI:** 10.1002/ece3.4434

**Published:** 2018-08-24

**Authors:** Rachel L. Moran, Muchu Zhou, Julian M. Catchen, Rebecca C. Fuller

**Affiliations:** ^1^ Program in Ecology, Evolution, and Conservation Biology University of Illinois at Urbana‐Champaign Champaign Illinois; ^2^ Department of Animal Biology University of Illinois at Urbana‐Champaign Champaign Illinois

## Abstract

Behavioral isolation is thought to arise early in speciation due to differential sexual and/or natural selection favoring different preferences and traits in different lineages. Instead, behavioral isolation can arise due to reinforcement favoring traits and preferences that prevent maladaptive hybridization. In darters, female preference for male coloration has been hypothesized to drive speciation, because behavioral isolation evolves before F1 inviability. However, as with many long‐lived organisms, the fitness of second‐generation hybrids has not been assessed because raising animals to adulthood in the laboratory is challenging. Of late, reinforcement of male preferences has been implicated in darters because male preference for conspecific females is high in sympatry but absent in allopatry in multiple species pairs. The hypothesis that reinforcement accounts for behavioral isolation in sympatry assumes that hybridization and postzygotic isolation are present. Here, we used genomic and morphological data to demonstrate that hybridization is ongoing between orangethroat and rainbow darters and used hybrids collected from nature to measure postzygotic barriers across two hybrid generations. We observed sex ratio distortion in adult F1s and a dramatic reduction in backcross survival. Our findings indicate that selection to avoid hybridization promotes the evolution of male‐driven behavioral isolation via reinforcement in this system.

## 1 INTRODUCTION

The increasing availability of genomic sequence data for nonmodel organisms has revealed that hybridization is surprisingly common between species (Abbott et al., 2013; Mallet, 2005). As hybridization has traditionally been thought of as a homogenizing force, a major question in evolutionary biology is how speciation can proceed in the face of gene flow (Bolnick & Fitzpatrick, 2007; Feder, Egan, & Nosil, 2012; Felsenstein, 1981; Harrison & Larson, 2014). Despite a contentious history, it is now recognized that hybridization can actually promote speciation through reinforcement, the process by which enhanced prezygotic isolation is favored in sympatry in response to postzygotic isolation (Coyne & Orr, 2004; Dobzhansky, 1937; Servedio & Noor, 2003). Reinforcement causes reproductive character displacement (RCD), whereby behavioral isolation between two species is heightened in sympatry compared to allopatry. Although multiple different evolutionary forces can lead to such a pattern (reviewed in Hoskin & Higgie, 2010), it is considered reinforcement when the mechanism underlying RCD is selection against hybridization (Pfennig & Pfennig, 2012). Empirical and theoretical research has indicated that reinforcement may be more common than previously thought (Hudson & Price, 2014; Yukilevich, 2012) and can both directly finalize speciation in sympatry and indirectly initiate speciation in allopatry (via cascade reinforcement; Ortiz‐Barrientos, Grealy, & Nosil, 2009).

Our goal here was to use genomic data to investigate a putative hybrid zone between two species of darters and to examine the strength of multiple postzygotic barriers between these species to test the hypothesis that reinforcement contributes to speciation in this system. The two focal species exhibit a pattern of behavioral isolation consistent with reinforcement of male mating preferences (i.e., male preference for conspecific females is high in allopatry) (Moran & Fuller, 2018). Whether or not postzygotic isolation is present is unknown. Previous studies have shown a lack of postzygotic isolation through the F1 larval stage (Hubbs & Strawn, 1957). However, the total strength of postzygotic isolation is frequently underestimated using F1 hybrid inviability as the sole measurement of postzygotic isolation (Lemmon & Lemmon, 2010; Wiley, Qvarnström, Andersson, Borge, & Saetre, 2009). This is particularly problematic because genetic incompatibilities can be masked in F1s due to effects of dominance (Coyne & Orr, 2004; Mallet, 2006), and maternal provisioning can reduce F1 inviability (Schrader & Travis, 2008). Accurate estimates of postzygotic isolation therefore require quantifying postzygotic barriers in F1 adults and in later generation hybrids, but this can be quite challenging in long‐lived and/or nonmodel organisms. Measuring the total strength of postzygotic isolation typically necessitates generating multiple generations of hybrid crosses and raising the offspring in the laboratory through the adult life stage. This can be logistically challenging. This study solves this problem by identifying F1 hybrids in nature and using them to generate second‐generation hybrids and measure postzygotic isolation.

Darters are a diverse group of stream fishes that have been characterized as a model system for the evolution of speciation via sexual selection. Behavioral isolation evolves before F1 larval inviability in darters (Martin & Mendelson, 2016b; Mendelson, 2003; Mendelson, Imhoff, & Iovine, 2006; Mendelson, Imhoff, & Venditti, 2007; Williams & Mendelson, 2014), and there are no known cases of complete F1 inviability through the fertilization and larval hatching stage, even between very distantly related species. The apparent rapid evolution of prezygotic isolation relative to postzygotic isolation in these fish has been attributed to female mate choice on species‐specific male color traits (Williams & Mendelson, 2010, 2011, 2013). However, recent research in a number of darter species has found that strong conspecific mate preferences are exhibited by males but such preferences are weak (or sometimes absent) in females, and that male coloration functions primarily in male–male competition rather than female mate choice (Martin & Mendelson, 2016a; Mendelson, Gumm, Martin, & Ciccotto, 2018; Moran & Fuller, 2018; Moran, Zhou, Catchen, & Fuller, 2017; Zhou & Fuller, 2016; Zhou, Loew, & Fuller, 2015). Thus, males may actually play a stronger role than females in maintaining species boundaries, despite the presence of traditional sex roles and extreme sexual dimorphism.

This study focuses on the rainbow darter *Etheostoma caeruleum* and the orangethroat darter *Etheostoma spectabile*. The orangethroat darter is a member of the *Ceasia* clade (also referred to as the orangethroat darter clade), which consists of 15 allopatrically distributed species. Time‐calibrated gene phylogenies estimate that species within the orangethroat clade last shared a common ancestor 6–7 million years ago (mya) (Bossu, Beaulieu, Ceas, & Near, 2013). The orangethroat darter clade and rainbow darters are classified together in the subgenus *Oligocephalus*. Divergence time between rainbow and orangethroat darters has been estimated at 22 mya (Near et al., 2011), but these species have very similar male color patterns, ecology, and mating behavior. Thirteen of the orangethroat clade species occur sympatrically with rainbow darters, and ancient hybridization events are evident from the presence of introgressed rainbow darter mitochondrial haplotypes in four orangethroat species (i.e., orangethroat darter *E. spectabile,* current darter *E. uniporum*, brooks darter *E. burri*, and buffalo darter *E. bison*; Ray, Lang, Wood, & Mayden, 2008; Bossu & Near, 2009). Molecular evidence also suggests that hybridization is ongoing between the rainbow darter and two species in the orangethroat darter clade (i.e., the buffalo darter and the current darter), as early‐generation hybrids have been documented in nature (Bossu & Near, 2013; Moran et al., 2017). However, the evolutionary consequences of hybridization in darters remain unexplored.

Recent studies have suggested that selection against interspecific interactions (i.e., mating and fighting) contribute to behavioral isolation between orangethroat and rainbow darters. In sympatric pairings between rainbow darters and five different orangethroat darter clade species, males have been shown to exert strong preferences for mating with conspecific females and fighting with conspecific males (Moran et al., 2017). Such preferences are absent in allopatric pairings of rainbow and orangethroat darters with similar divergence times to the sympatric pairings (Moran & Fuller, 2018). This pattern is consistent with both RCD in male mating preferences and divergent agonistic character displacement (ACD) in male fighting preferences. Divergent ACD occurs when selection against interspecific aggressive interactions leads to the evolution of enhanced bias against fighting with heterospecifics in sympatry (Grether, Losin, Anderson, & Okamoto, 2009). In addition, behavioral experiments simulating secondary contact between multiple allopatric orangethroat darter clade species revealed that males also prefer to mate and fight with conspecifics over other orangethroat species, but only when they occur sympatrically with rainbow darters (Moran & Fuller, 2018). This suggests that RCD and ACD in sympatry between orangethroat and rainbow darters may have cascading effects by incidentally initiating trait evolution and male‐driven behavioral isolation among lineages within the orangethroat darter clade. It is surprising that studies have consistently failed to detect female preferences in orangethroat and rainbow darters for varying components of male color pattern within or between species (Fuller, 2003; Moran et al., 2017; Pyron, 1995; Zhou et al., 2015).

Whether reinforcement is causing the pattern of RCD in male mating preferences in orangethroat and rainbow darters remains uncertain. Previous investigations into postzygotic barriers between orangethroat and rainbow darters have been limited to examining F1 larval survival and have found no evidence of hybrid inviability through this life stage (Bossu, 2012; Bossu & Near, 2013; Hubbs, 1967; Hubbs & Strawn, 1957; Linder, 1958). Here, we use phenotypic and genomic data to confirm that hybridization is ongoing between the orangethroat darter and the rainbow darter and then investigate postzygotic isolation between these species using both laboratory‐generated and wild‐caught hybrids. We test for inviability, sex ratio distortion, sterility, and mating behavioral abnormalities in F1 hybrids, and inviability in backcross hybrids. This represents the most thorough investigation to date into postzygotic isolation in darters. By utilizing natural hybrids, we were able to reveal that postzygotic isolation is much higher than previously thought. We present evidence that hybridization is ongoing and that it is maladaptive, providing critical support for the hypothesis that male‐driven behavioral isolation has evolved via reinforcement (and cascade reinforcement) in these species. More general, these results contribute to our understanding of the evolution of concurrent RCD and ACD in male mating preferences and fighting biases.

## 2 METHODS

### 2.1 Laboratory F1 hybrid cross viability

We first created F1 hybrids in the laboratory. Adult orangethroat and rainbow darters were collected from two adjacent tributaries of the Vermillion River (Champaign Co., Illinois; Supporting Information Table S1) using a kick seine in April and May 2012. Fish were transported back to the University of Illinois at Urbana‐Champaign. Crosses were performed by hand‐stripping eggs from a single female into a petri dish filled with water from their native stream and subsequently hand‐stripping sperm from a single male onto the eggs. Afterward, the water in the petri dish was gently swirled for 1 min to mix the eggs and sperm. Each clutch of eggs was transferred to a separate plastic tub filled with water that was treated with methylene blue (to prevent fungal growth) and stored in an incubator set to 11°C and a 11:14‐h light:dark cycle.

Unique male–female pairs were used as parents in each replicate cross. We performed F1 crosses in both direction and “purebred” control crosses with both parental species, with 10–14 replicates per cross type (Table 1). The eggs from each replicate were checked daily for development. As fry hatched, they were transferred to a larger tub in the incubator and fed live brine shrimp nauplii every other day. Fry were transferred out of the incubator and into 19 L and 38 L aquaria at approximately 3 weeks posthatching. Aquaria were maintained at 19°C, and the photoperiod was set to mimic natural daylight hours. After transfer to the aquaria, fish were fed daily ad libitum with frozen daphnia and frozen bloodworms.

**Table 1:** Mean (±standard error) number of total eggs stripped, eggs fertilized, eggs hatched, and fry that survived to 10 months of age in the purebred crosses and F1 hybrid crosses

Cross	Number of eggs stripped	Number of eggs fertilized	Number of eggs hatched	Number of fry survived 10 months
♀ O × ♂ O	67.4 ± 9.2 (*n* = 14)	30.6 ± 12.3 (*n* = 11)	24.6 ± 13.0 (*n* = 10)	6.5 ± 0.6 (*n* = 6)
♀ R × ♂ R	76.0 ± 14.2 (*n* = 12)	25.7 ± 9.9 (*n* = 11)	22.4 ± 10.7 (*n* = 9)	5.8 ± 2.2 (*n* = 8)
♀ O × ♂ R	92.8 ± 16.0 (*n* = 10)	23.9 ± 9.7 (*n* = 10)	14.5 ± 5.3 (*n* = 8)	3.8 ± 0.7 (*n* = 5)
♀ R × ♂ O	80.2 ± 12.1 (*n* = 12)	30.6 ± 7.5 (*n* = 11)	22.1 ± 6.8 (*n* = 9)	6.0 ± 1.1 (*n* = 9)

*Note*

O: orangethroat darter; R: rainbow darter.

We measured fertilization success (proportion of eggs that developed pigmented eyes), hatching success (proportion fertilized eggs that yielded free‐swimming fry), and larval survival (proportion of hatched eggs that survived to 10 months) of each family. In addition, to determine whether the mean sex ratio of each cross type deviated from the expected 1:1, we measured the sex ratio of each family after 22 months. By this time, all fish exhibited sexually dimorphic coloration.

All statistical analyses were conducted in R (version 3.4.0). We asked whether each viability metric (fertilization success, hatching success, and larval survival) varied among cross types at the family level using generalized linear models (GLMs), with the viability metric as the independent variable and cross type as the dependent variable. We conducted these analyses using the *glm* function of the *stats* package and specified a quasibinomial distribution with logit link function to account for overdispersion in the data. We used the *Anova* function of the *car* package (Fox, 2007) to generate type II analysis of deviance tables and F tests. We also used one‐sample Student's *t* tests using the *t.test* function of the *stats* package to test whether the proportion of male offspring in a clutch differed from the expected 0.50 in each cross type.

### 2.2 Backcross viability using wild‐caught F1 hybrids

Here, we backcrossed wild‐caught F1 hybrid males to orangethroat and rainbow darter females. We used F1 individuals collected from a natural hybrid zone as parents in backcrosses rather than using laboratory‐generated F1s because at 2 years of age most of our laboratory‐raised orangethroat darters and F1 hybrids failed to engage in mating behavior and females were not gravid. This was not completely unexpected, as orangethroat and rainbow darters can take up to 3 years to reach sexual maturity in the laboratory (R. Moran, pers. obs.). However, it is also possible that the artificial laboratory rearing environment lacked a critical cue to trigger the onset of spawning. We therefore only used wild‐caught fish for backcrosses.

**Figure 1:**
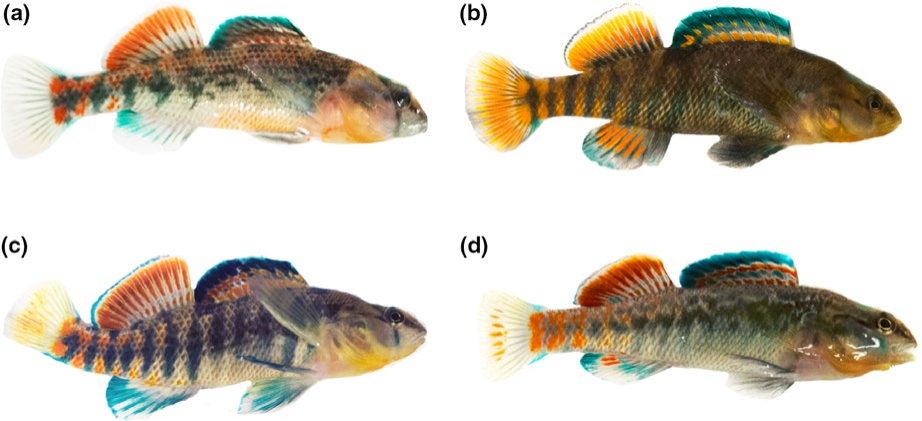
(a) Orangethroat darter and (b) rainbow darter males showing color pattern typical of these species. Orangethroat darters lack the red coloration that is present on the caudal and anal fin in rainbow darters. (c) Wild‐caught orangethroat × rainbow darter F1 hybrid male and (d) laboratory‐generated orangethroat × rainbow darter F1 hybrid male showing color pattern characteristics that are combinations of both parental species

We collected adult male and female orangethroat and rainbow darters and F1 hybrid males from three tributaries of the Vermillion River (Champaign Co., Illinois; Supporting Information Table S1) in April 2016. We chose to use F1 hybrid males (rather than females) to measure backcross viability because preliminary analyses of our F1 laboratory crosses revealed that: (a) hybrid males are diagnosable due to their color pattern intermediacy between the parental species (see below) (Figure 1), and (b) the sex ratio of F1 hybrid clutches is dramatically skewed toward males, which suggests that F1 females may be quite rare in natural populations (see below). We confirmed our initial classification of wild‐caught fish as orangethroats, rainbows, or hybrids using multivariate phenotypic analyses and genetic sequencing (see below).

We conducted four cross types with six replicates each. We conducted backcrosses in both directions between the wild‐caught F1 males and parental species females and conducted “purebred” control crosses with both parental species (Table 2). Crosses were conducted in breeding aquaria filled with 5–7 cm of naturally colored aquarium gravel, and fluorescent lighting was provided that mimicked the natural photoperiod. Fish were fed frozen bloodworms ad libitum each day.

**Table 2:** Mean (±standard error) number of total eggs collected, eggs fertilized, eggs hatched, and fry that survived to the independently feeding stage in purebred crosses and backcrosses (*n* = 6 each)

Cross	Number of Eggs collected	Number of Eggs fertilized	Number of Eggs hatched	Number of Fry survived to feeding
♂ O × ♀ O	92.33 ± 14.83	82.00 ± 12.41	61.67 ± 9.90	56.17 ± 8.18
♂ R × ♀ R	35.50 ± 5.23	31.17 ± 3.61	21.67 ± 3.19	21.00 ± 3.36
♂ H × ♀ O	88.00 ± 30.46	19.33 ± 14.81	10.33 ± 10.14	6.00 ± 5.80
♂ H × ♀ R	23.17 ± 5.76	8.33 ± 2.39	2.50 ± 1.77	2.17 ± 1.60

*Note*

O: orangethroat darter; R: rainbow darter; H: F1 hybrid.

To generate backcrosses, a hybrid male was rotated daily between two 37.9‐L breeding aquaria, one of which contained an orangethroat darter female and the other a rainbow darter female. We used a small dip net to rotate hybrid males from one backcross breeding aquarium to the other every day at noon for 14 consecutive days, so that hybrid males spent 7 days with each of the two parental females. Eggs were collected from each breeding aquarium immediately after the hybrid male was transferred to the other parental female's breeding aquarium. Eggs were collected each day for 7 days from each purebred parental pair. Purebred parental males were also moved from their breeding aquaria to a separate holding tank for 10 min once a day. During this time, the eggs were collected from the breeding aquaria. Eggs collected from each breeding aquarium were kept together in a 1‐L container and maintained as described in the previous section. For each cross, we measured offspring viability at three developmental stages: the proportion of eggs that were fertilized, the proportion of fertilized eggs that survived to hatching, and the proportion of hatched fry that survived to the larval feeding stage (approximately 3 days posthatching).

We first asked whether the three measures of viability varied as a function of cross following the same methodology as described above for the F1 crosses. The *glht* function of the *multcomp* R package (Hothorn, Bretz, Westfall, & Heiberger, 2017) was used to make post hoc pairwise comparisons between cross types. We also asked whether females used in backcrosses were as likely to produce eggs as those used in the purebred parental crosses. We conducted two separate Mann–Whitney *U* tests to determine whether the total number of eggs produced by orangethroat and rainbow darter females differed depending on the identity of the male that they were paired with (i.e., hybrid male or purebred conspecific male). Female standard length did not differ between species (mean ± *SE*: orangethroat* = *61.45 ± 1.43 mm, *n* = 12; rainbow* = *57.97 ± 1.66 mm, *n* = 12; two‐sample *t* test: *t*_21.52_ = 1.52, *p* = 0.14), and male standard length did not differ among groups (mean ± *SE*: hybrids = 65.4 ± 3.2 mm, *n* = 6; orangethroat* = *68.6 ± 3.0 mm, *n* = 6; rainbow* = *68.0 ± 1.6 mm, *n* = 6; ANOVA: *F*_2,15_ = 0.34, *p* = 0.72).

### 2.3 Wild‐caught F1 hybrid male mating and competitive behavior

Both orangethroat and rainbow darters congregate in shallow, gravel riffles of headwater streams during the spring breeding season. Males attempt to guard females by chasing off male competitors and flaring their fins in threat displays. Once a female is ready to spawn, she will perform a nosedig into the gravel and bury herself in the substrate. If multiple males are near the female at this time, male fighting will escalate. One to several males will then attempt to spawn with the female (Fuller, 2003; Winn, 1958).

We conducted two types of behavioral trials to examine mating behavior of wild‐caught F1 hybrid males: dichotomous male choice trials and male competition trials. These behavioral trials used the same wild‐caught F1 hybrid males as the backcross experiment described above but used different orangethroat and rainbow darter individuals from the same drainage (i.e., Vermillion River, Champaign Co., Illinois). Previous behavioral studies have shown that orangethroat and rainbow darter males from this drainage exhibit strong preferences for mating and fighting with members of their own species over the other (Moran & Fuller, 2018; Moran et al., 2017; Zhou & Fuller, 2014). Here, our goal was to ask whether hybrid males show any preference for mating or fighting with members of either parental species. Each behavioral trial involved three fish in a 37.9‐L test aquarium positioned under a fluorescent light and filled with 5–7 cm of naturally colored gravel.

For the dichotomous male mate choice trials, a hybrid male was joined by a female orangethroat and a female rainbow darter (*n* = 6). This allowed us to observe whether hybrid males would choose to pursue either female, and, if so, whether they exhibited a preference for females of either species. We split each trial into 60 30‐s blocks. We scored the number of 30‐s blocks in which the male was within one body length of each female for a minimum consecutive time of 5 s (Moran & Fuller, 2018; Zhou et al., 2015). We used one‐sample Student's *t* tests with the *t.test* function of the *stats* package in R to test whether the proportion of blocks that the male spent pursuing the orangethroat darter female (vs. the total number of blocks spent pursuing either female) differed from the expected 0.50 in each trial.

For the male competition trials, a hybrid male was joined by a male–female pair that were either both orangethroat or both rainbow darters. The goal of these trials was to measure male–male aggressive behavior, but a female was included to elicit male competitive behavior. Each hybrid male participated in two consecutive competition trials, one in which he was joined by an orangethroat darter pair (*n* = 6) and one in which he was joined by a rainbow darter pair (*n* = 6). Thus, each hybrid male was involved in a total of three behavioral trials: one dichotomous male choice trial and two male competition trials. Hybrid males experienced these trial types in random order. Unique purebred fish were used in each trial. We measured hybrid male aggressive behavior by counting the number of attacks (chasing and biting) and fin flares (male threat displays) that the hybrid male performed toward the purebred male in each trial (Moran et al., 2017; Zhou et al., 2015). We asked whether the number of attacks and fin flares that hybrid males directed toward males of the two purebred species differed. We performed GLMs with a negative binomial distribution and logit link function using the *glm.nb* function of the *MASS* package in R (Ripley, Venables, Bates, Hornik, & Gebhardt, 2017). We performed separate GLM analyses that included the number of male aggressive behaviors (fin flares or attacks) performed in each trial as the dependent variable, and the identity of the purebred species pair in the trial (orangethroat or rainbow) as the independent variable.

### 2.4 Morphological and histological analyses of testes

To further investigate potential F1 hybrid male sterility, we examined the testes of the six hybrid males and the 12 parental males (six orangethroat and six rainbow darters) that were used in the backcross experiment. Males were euthanized with an overdose of buffered MS‐222. We performed gross and histological analyses to compare the testes of the hybrid and purebred males. Testes from each male were fixed in 10% buffered formalin, embedded in paraffin wax, and sectioned. Four‐micrometer sections were stained with hematoxylin and eosin and were visually inspected for signs of normal spermatogenesis.

### 2.5 Color analyses

We used digital photographs to perform multivariate phenotypic analyses of wild‐caught orangethroat and rainbow darter males, wild‐caught putative F1 hybrid males, and laboratory‐generated F1 hybrid males. Our aim was to quantify differences in male color pattern in purebred males and hybrid males, and to statistically verify that hybrid color pattern is distinct and intermediate between purebred species. Such a finding would support our classification of wild‐caught F1 hybrid males used in backcross experiments.

We chose to focus on components of male color pattern that differ between the parental species. Superficially, the red and blue banding pattern of orangethroat and rainbow darters looks quite similar, but these species differ in several key ways. Figure 1a,b illustrates the differences in male color pattern characteristics between orangethroat and rainbow darters, the most obvious of which are lateral side banding pattern and coloration, anal fin coloration, and caudal fin coloration. Our observations of laboratory‐generated and wild‐caught F1 hybrid males indicate that hybrids appear to exhibit combinations of both purebred species’ color patterns (Figure 1c,d).

We measured 36 male color pattern variables (i.e., 27 RGB variables and nine color proportion variables; see Supporting Information for additional details) in the wild‐caught hybrid males, orangethroat males, and rainbow males (*n* = 6 each) used in backcross experiments, and in six laboratory‐generated F1 hybrid males (which each came from unique families; three from ♀ rainbow × ♂ orangethroat crosses, three from ♀ orangethroat × ♂ rainbow crosses). We performed linear discriminant analysis (LDA) on the male color pattern data with group (i.e., orangethroat*,* rainbow, wild‐caught hybrid, or laboratory‐generated hybrid) as the predictor variable using the *lda* function of the *MASS* package in R (Ripley et al., 2017). LDA identifies combinations of independent variables that maximize separation between dependent variables (Mika, Ratsch, Weston, Scholkopf, & Mullers, 1999). Thus, groups with more disparate loadings for a given linear discriminant (LD) can be inferred to be more distinct from one another in multivariate signal space. To ask whether male color pattern differs significantly between groups, we conducted multivariate analysis of variance (MANOVA) using the *manova* function of the *stats* package in R. Color measurements served as the independent variables and group served as the dependent variable.

### 2.6 Genotyping wild‐caught purebred and hybrid fish

To further verify the purebred or hybrid classification of all fish used in the backcross experiment (42 fish total), we performed single‐digest restriction site‐associated DNA sequencing (RADseq). DNA was isolated from skin and muscle tissue using a modified Puregene protocol. Samples were normalized to a concentration of 15 ng/μL in 50 μL 1× TE. RADseq library preparation with the restriction enzyme *Sbf*I was performed by Floragenex (Eugene, OR, USA), following the methods of Baird et al. (2008). The resulting RADseq library was sequenced as single‐end 100 bp reads on two lanes on an Illumina HiSeq 4000 machine.

Sequencing resulted in a total of 37,007,596 reads across the 42 individuals, with a mean ± *SE* of 881,133 ± 197,553 reads per individual. We used the Stacks (v2.0Beta9; Catchen, Amores, Hohenlohe, Cresko, & Postlethwait, 2011; Catchen, Hohenlohe, Bassham, Amores, & Cresko, 2013) *process_radtags* program to demultiplex samples, remove barcodes, and remove reads of low quality or with ambiguous barcodes. This resulted in a total of 36,232,000 retained reads, which were then supplied to the *denovo_map* pipeline in Stacks to construct a catalog of loci and call SNPs. A minimum of three identical reads were required for each locus (‐m 3), with a maximum of three mismatches between loci in each individual (‐M 3), and a maximum of two mismatches between loci to be added to the catalog (‐n 2). This resulted in a catalog of 63,891 variant sites across 123,901 loci, representing a total of 11,308,200 sites across the genome. The mean ± *SE* depth of coverage was 23 ± 3× per individual.

The *populations* program in Stacks was used to generate population genetic statistics and to filter loci for analysis of genetic ancestry in Structure (Pritchard, Stephens, & Donnelly, 2000). We used *populations* to select loci that were present in all three groups (i.e., orangethroats, rainbows, and putative hybrids) (‐p 3) and in at least 50% of the individuals within a group (‐r 0.5), with a minimum minor allele frequency of 3%. This filtering resulted in 1,897 SNPs across 1,351 loci (representing a total of 123,472 sites across the genome) for the set of 42 total individuals (six hybrids, 18 rainbows, 18 orangethroats). To make comparisons between hybrids and parental species, we used *populations* to calculate statistics of genetic differentiation between groups, including SNP‐based AMOVA F_ST_ (Weir, 1996) and haplotype‐based Φ_ST_ (analogous to F_ST_; Excoffier, Smouse, & Quattro, 1992) and D_EST_ (Jost, 2008). Unlike *F*_ST_ and Φ_ST_, *D*_EST_ is not sensitive to the level of heterozygosity within groups. To obtain an absolute measure of pairwise divergence, we used DnaSP (v6.10.03) (Rozas, Sanchez‐DelBarrio, Messeguer, & Rozas, 2003) to calculate the average number of nucleotide differences between groups (*D*_xy_). To measure the level of genetic diversity within groups, we obtained estimates of nucleotide diversity (π), heterozygosity, the percent of polymorphic sites, and the number of private alleles from *populations*.

In the event that more than one SNP was present at a given RAD locus, we only used first SNP for Structure analyses by supplying the –write_single_snp flag to *populations*. This resulted in 1,073 unlinked SNPs that were output in Structure file format. To infer the number of distinct genetic clusters present in the data, we ran Structure with the ancestry model that allowed for admixture and a burn‐in length of 50,000 followed by 150,000 MCMC repetitions. We performed 20 runs for values of *K* (i.e., genetic clusters) from 1 to 5 and inferred the optimal value of *K* using the Evanno method (Evanno, Regnaut, & Goudet, 2005) in Structure Harvester (Earl & vonHoldt, 2012). Preliminary analyses confirmed the presence of two distinct genetic clusters in the dataset, one corresponding to orangethroat darters and the other to rainbow darters (see Results).

To infer the proportion of ancestry associated with orangethroat versus rainbow darters in each hybrid male, we also calculated the hybrid index in GenoDive (v2.0b27) (Meirmans & Van Tienderen, 2004) following the method of Buerkle (2005). The hybrid index is a maximum‐likelihood estimate of the proportion of alleles in a hybrid individual that originated from one parental species versus the other. We imported the Structure file containing genotype data for 1,073 SNPs across all 42 individuals into GenoDive. For a given hybrid individual, a hybrid index closer to 1 would indicate allele frequencies more similar to that of orangethroat darters, and a hybrid index closer to 0 would indicate allele frequencies more similar to that of rainbow darters.

## 3 RESULTS

### 3.1 Laboratory F1 hybrid cross viability

Fertilization success, hatching success, and larval survival did not differ between F1 hybrid clutches and the “purebred” parental species clutches (fertilization success: *F*_3,39_ = 0.51, *p* = 0.68; hatching success: *F*_3,39_ = 0.04, *p* = 0.99; fry survival: *F*_3,32_ = 0.31, *p* = 0.82; Table 1, Supporting Information Figure S1). Fertilization success varied greatly across replicate clutches but averaged less than 50% for all cross types. There were five clutches in which none of the eggs developed, possibly due to them being unripe or overly ripe (Moran, Soukup, Zhou, & Fuller, 2018). Excluding these five crosses from the analysis did not qualitatively change the results. On average, over 50% of fertilized eggs hatched. Mortality was minimal between 10 and 22 months. Eight hybrids and three purebred fish died during this period, but most deaths could be attributed to husbandry issues (e.g., tank filter failure).

**Figure 2:**
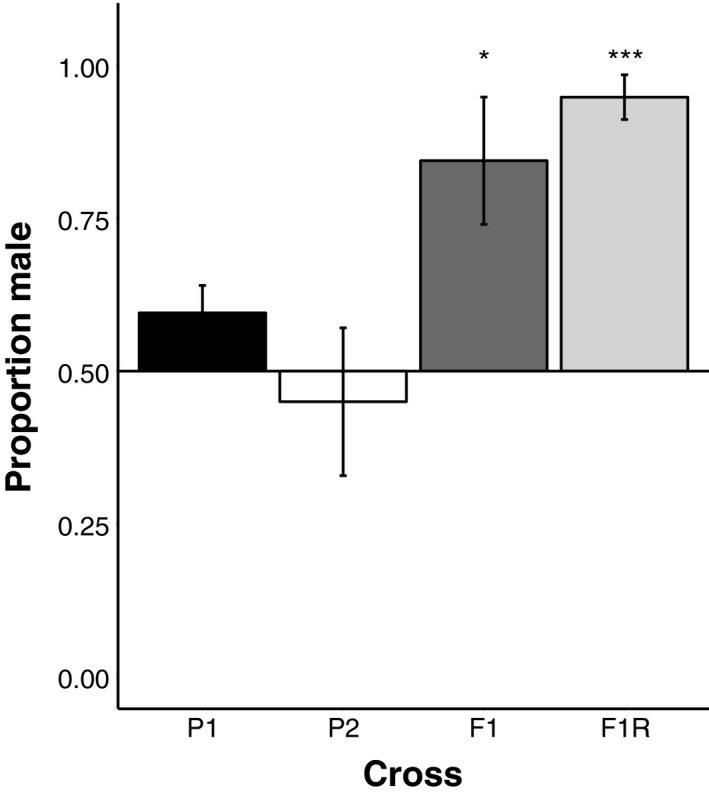
Mean proportion (±standard error) of male offspring in the parental crosses and F1 hybrid crosses at 10 months of age. The deviation from a mean of 0.50 male offspring (i.e., a 1:1 male:female sex ratio) is depicted for each cross type (* = *p* < 0.05, *** = *p* < 0.001). P1 = ♀ orangethroat × ♂ orangethroat (*n* = 6), P2 = ♀ rainbow × ♂ rainbow (*n* = 8), F1 = ♀ orangethroat × ♂ rainbow (*n* = 5), F1R = ♀ rainbow × ♂ orangethroat (*n* = 9)

In both F1 hybrid crosses, the sex ratio of the offspring was significantly skewed toward males (♀ orangethroat × ♂ rainbow: mean ± *SE* proportion male = 0.844 ± 0.104, *t*_5_ = 3.30, *p* = 0.02; ♀ rainbow × ♂ orangethroat: mean ± *SE* = 0.948 ± 0.037, *t*_6_ = 12.26, *p* < 0.00001) (Figure 2). Only four of the 13 F1 hybrid families included females at 22 months. A total of six of 65 F1 hybrids were female. The sex ratio did not differ from the expected 1:1 frequency in purebred crosses (♀ orangethroat × ♂ orangethroat: mean ± *SE* = 0.594 ± 0.045, *t*_5_ = 2.13, *p* = 0.09; ♀ rainbow × ♂ rainbow: mean ± *SE* = 0.450 ± 0.121, *t*_8_ = −0.41, *p* = 0.69) (Figure 2). Eleven of 15 purebred families contained offspring of both sexes at 22 months of age. The total number of offspring per clutch at 22 months did not differ between hybrid and purebred crosses (*F*_1,26_ = 0.18, *p* = 0.68).

### 3.2 Backcross viability using wild‐caught F1 hybrids

**Figure 3:**
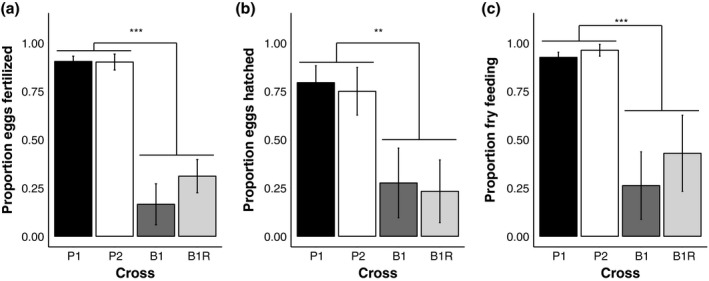
Mean proportion (±standard error) of (a) eggs collected that were fertilized, (b) fertilized eggs that hatched, and (c) hatched fry that survived to the independently feeding stage (approximately 3 days posthatching) in the parental crosses and backcrosses (*n* = 6 each). Significance levels are indicated for post hoc comparisons of purebred crosses and backcrosses (** = *p* < 0.01, *** = *p* < 0.001). P1 = ♀ orangethroat × ♂ orangethroat, P2 = ♀ rainbow × ♂ rainbow, B1 = ♀ orangethroat × ♂ F1 hybrid, B1R = ♀ rainbow × ♂ F1 hybrid

Backcrosses suffered higher levels of inviability compared to “purebred” orangethroat and rainbow darter crosses across all three measures of offspring viability (proportion of eggs collected that were fertilized: *F*_3,20_ = 19.02, *p* < 0.00001; proportion of fertilized eggs that hatched: *F*_3,20_ = 3.47, *p* < 0.05; proportion of hatched eggs that survived to the feeding larval stage: *F*_3,20_ = 6.95, *p* < 0.01; Table 2, Figure 3). Fertilized eggs were collected in 10 of 12 (83%) of the hybrid male crosses; one hybrid male × orangethroat darter female backcross replicate and one hybrid male × rainbow darter female backcross replicate yielded no fertilized eggs. All purebred crosses produced fertilized eggs. Cumulative survival across all developmental stages was 10× higher in purebred crosses than backcrosses (Table 2). We did not observe any asymmetry in backcross viability: Backcross to both parental species showed equally low levels of viability at each of the three developmental stages measured (Figure 3). Parental crosses also did not differ from one another in viability at any stage (Figure 3).

Orangethroat and rainbow darter females used in the crosses produced a similar number of eggs regardless of whether they were paired with a hybrid or a purebred conspecific male (Table 2; orangethroat backcross versus purebred cross: Mann–Whitney *U* test: *U* = 18, *n* = 12, *p* = 1.00; rainbow backcross versus purebred cross: Mann–Whitney *U* test: *U* = 8, *n* = 12, *p* = 0.13). In general, rainbow darter females laid fewer, larger eggs compared to orangethroat females, which laid a larger number of smaller eggs (R. Moran, pers. obs.). We observed that female orangethroat darters laid two to three times more eggs than female rainbow darters of equivalent size during the duration of this experiment. However, the proportion of offspring surviving through each developmental stage did not differ between species (Figure 3; Table 2).

### 3.3 Wild‐caught F1 hybrid male mating and competitive behavior

Previous behavioral studies in orangethroat and rainbow darters have shown that males of both species exhibit strong preferences for pursuing females of their own species and preferentially direct aggressive behaviors toward males of their own species (Moran & Fuller, 2018; Moran et al., 2017). In contrast, we observed no indication of assortative mating preferences in the wild‐caught F1 hybrid males in our dichotomous male choice trials. Hybrid males did not preferentially pursue one purebred species of female over the other (Supporting Information Figure S2a; *t*_5_ = −0.12, *n* = 6, *p* = 0.91). In the same way, hybrid males did not preferentially bias their aggression toward orangethroat or rainbow darter males in the male competition trials. Hybrid males performed a similar number of fin flares (*X* ^2 = ^0.51, *n* = 6, *p* = 0.48; Supporting Information Figure S2b) and attacks (*X* ^2 = ^0.13, *n* = 6, *p* = 0.72; Supporting Information Figure S2c) toward males of both parental species. In addition, all orangethroat and rainbow darter males engaged in aggressive interactions with the hybrid males.

### 3.4 Morphological and histological analyses of testes

Gross examination determined that all hybrid males possessed normally developed testes, compared to the purebred orangethroat and rainbow darter males. Comparative histological analysis of the hybrid and purebred male testes revealed that the testes of all males examined contained mature spermatids, and no obvious irregularities in spermatogenesis were observed. Figure S3 (Supporting Information) shows representative images of testes histology for an orangethroat darter male, a rainbow darter male, and two wild‐caught F1 hybrid males.

### 3.5 Color analyses

**Figure 4:**
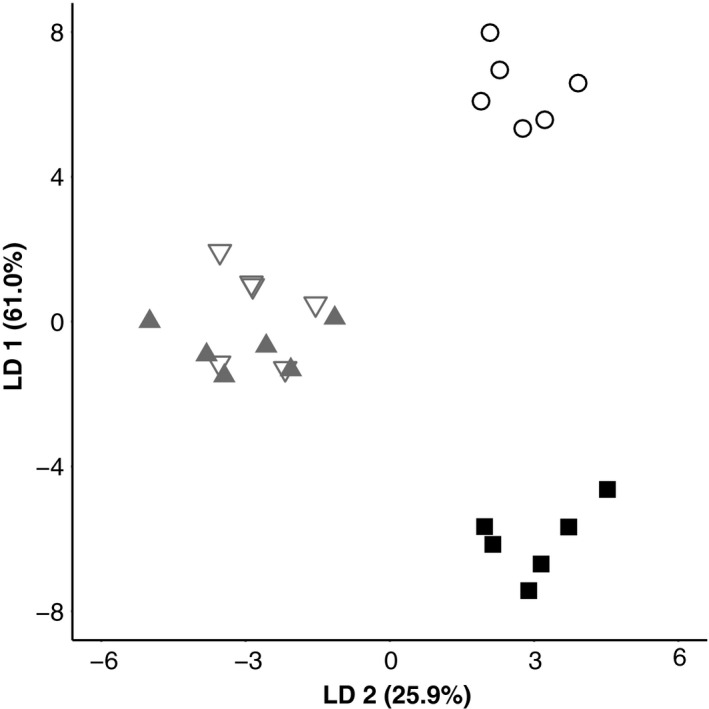
Biplot of the first two linear discriminant (LD) axes from the male color pattern LDA of wild‐caught orangethroat (■), wild‐caught rainbow (○), wild‐caught F1 hybrid males (▽), and laboratory‐generated F1 hybrid males (▲)

The LDA of male color pattern for orangethroat, rainbow, and F1 hybrid males simplified the multivariate color dataset of 27 RGB variables and nine color proportion variables into three LDs. The first two LDs explained a combined total of nearly 87% of the variance in coloration between groups. We visualized the differences in male color pattern among groups in two‐dimensional signal space by plotting scores for LD 1 versus LD 2 for each individual (Figure 4). Orangethroat, rainbow, and F1 hybrid individuals formed tight and well‐separated clusters. There was almost complete overlap between the clusters containing the laboratory‐raised and wild‐caught F1 hybrid males. Furthermore, hybrid individuals occupied a signal space intermediate between both purebred species along the axis corresponding to LD 1 (Figure 4, Supporting Information Figure S4).

The color proportion measurements had larger LD coefficients compared to the RGB measurements across all three LDs, indicating that the proportion of red and blue coloration on the body and fins is better predictors of group membership than RGB values (Supporting Information Table S2). We therefore used the color proportion measurements for subsequent analyses. There was a significant difference in male color pattern between orangethroat, rainbow, and hybrid males (MANOVA: Pillai's trace = 2.33, *F*_3,20_ = 5.40, *p* < 0.000001). Male color pattern did not differ between the laboratory‐generated and wild‐caught F1 hybrid males (MANOVA: Pillai's trace = 0.95, *F*_1,10_ = 4.22, *p* = 0.21).

### 3.6 Genotyping wild‐caught purebred and hybrid fish

As expected, notably higher levels of genetic diversity were observed within the hybrid group compared to either parental species (Table 3). Nucleotide diversity (*π*) and heterozygosity were generally low in both parental species, but higher in rainbow darters compared to orangethroat darters. In the hybrid fish, *π* was 7.6× higher compared to orangethroat darters and 6.1× higher compared to rainbow darters. In the same way, heterozygosity was 9.8× higher in hybrids compared to orangethroat darters and 5.9× higher in hybrids compared to rainbow darters. The number of private alleles was also an order of magnitude lower in the hybrid group compared to either parental species (Table 3), which is to be expected in F1 hybrids that share half of their alleles with each parental species.

**Table 3:** Measurements of genetic diversity within groups (i.e., hybrids, rainbow darters, and orangethroat darters) for all 123,472 sites across 1,351 loci

Group	*n*	% Poly	# Priv	*π*	*H*_OBS_	*H*_EXP_
Hybrids	6	1.2636	12	0.0061	0.0059	0.0052
Rainbow	18	0.5387	212	0.0010	0.0010	0.0012
Orangethroat	18	0.3694	103	0.0008	0.0006	0.0009

*Note*

% Poly: percent polymorphic sites; # Priv: number of private alleles; π: nucleotide diversity; *H*_OBS_: observed heterozygosity; *H*_EXP_: expected heterozygosity.

Patterns of genetic differentiation between groups also supported our classification of hybrid individuals. The SNP‐based F_ST_ was lower compared to the haplotype‐based Φ_ST_ and D_EST_, but all three measurements of genetic differentiation between groups indicated a high degree of differentiation between orangethroat and rainbow darters, with estimates ranging between 0.689 and 0.808 (Table 4). As expected, comparisons between hybrids and orangethroat darters and between hybrids and rainbow darters revealed lower levels of differentiation. The average number of nucleotide substitutions per site (*D*_xy_) was 0.01 between orangethroat and rainbow darters. *D*_xy_ between hybrids and each of the two parental species was 0.005, exactly half of that between the parental species.

**Table 4:** Measurements of genetic differentiation and divergence between groups (i.e., hybrids, rainbow darters, and orangethroat darters)

Comparison	*F*_ST_	Φ_ST_	*D*_EST_	*D*_XY_
Hybrids—rainbows	0.327	0.495	0.305	0.005
Hybrids—orangethroats	0.315	0.454	0.271	0.005
Rainbows—orangethroats	0.689	0.792	0.808	0.010

*Note*

The SNP‐based fixation statistic (*F*_ST_) was calculated using 1,897 variant sites (SNPs). The haplotype‐based fixation statistics (Φ_ST_, *D*_EST_) and the average number of nucleotide substitutions per site (*D*_XY_) were calculated using 123,472 sites across 1,351 loci.

**Figure 5:**
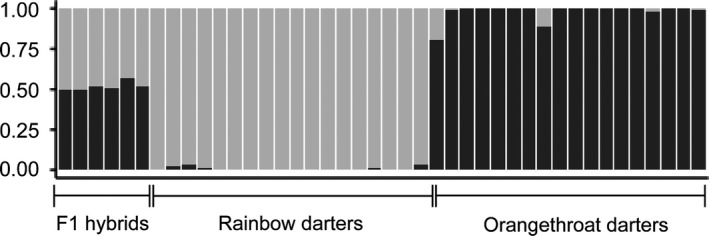
Probability of membership to the rainbow darter cluster (light gray) or orangethroat darter cluster (dark gray) for each individual used in the backcross experiment. Structure analysis was conducted using 1,073 SNPs with the number of clusters (*K*) set to 2

The Structure analysis of 1,073 SNPs present in the set of 42 individuals used in the backcross experiment revealed an optimal *K* of 2 according to the Evanno method implemented in Structure Harvester (Supporting Information Table S3). As with the color analyses, the genetic analyses confirmed our original diagnosis of the wild‐caught orangethroat darters, rainbow darters, and F1 hybrid males that were used in the backcross experiment (Figure 5). With *K* set to 2, the 18 orangethroat darter individuals were assigned 98% membership to Cluster 1, and the 18 rainbow darter individuals were assigned 99% membership to Cluster 2. The assignments of the six hybrid males were split between clusters and averaged 53% membership to the orangethroat cluster and 47% membership to the rainbow cluster. The hybrid index scores calculated for the hybrids yielded qualitatively similar results; the maximum‐likelihood estimate for the proportion of orangethroat darter ancestry in each hybrid male ranged from 0.501 to 0.566 (Supporting Information Table S4).

## 4 DISCUSSION

Here, we tested the hypothesis that reinforcement promotes the previously documented pattern of enhanced male mating preferences for conspecific over heterospecific females in sympatry compared to allopatry (i.e., RCD) in orangethroat and rainbow darters (Moran & Fuller, 2018). Reinforcement occurs when selection to avoid maladaptive hybridization favors divergence in mating signals and/or associated preferences in sympatry between two species (Coyne & Orr, 2004; Servedio & Noor, 2003). We used morphological and genomic data to show that hybridization is ongoing between orangethroat and rainbow darters. We then used natural and laboratory‐generated hybrids to measure multiple components of postzygotic isolation. Our results suggest that there is a fitness consequence associated with hybridization in these species. This drastically changes how we think about speciation in darters, one of the most diverse groups of vertebrates in North America. Below we discuss the unexpectedly high degree of postzygotic isolation that we observed between orangethroat and rainbow darters and its implications for male‐driven speciation via reinforcement and cascade reinforcement.

### 4.1 Patterns of F1 and backcross hybrid inviability

We found high levels of postzygotic isolation between orangethroat and rainbow darters in the form of multiple isolating barriers spanning across hybrid life stages and generations. This system was previously thought to lack substantial postzygotic isolation among species due to high survival of F1 larvae compared to purebred crosses (Hubbs, 1959; Hubbs & Strawn, 1957). Our results corroborated these previous findings. Clutches resulting from F1 crosses did not exhibit reduced fertilization, hatchability, or survival through adulthood compared to purebred crosses. However, we did observe dramatically distorted sex ratios in F1 crosses. Heterospecific crosses in both directions were heavily skewed toward males. Clutches from purebred crosses did not deviate from a 1:1 sex ratio, and most natural darter populations have also been shown to maintain 1:1 sex ratios in adults (Page, 1983). Whether the male‐skewed sex ratio in F1 hybrids creates selection favoring assortative mating and behavioral isolation in areas of sympatry is unclear. Such a scenario may be present in *Neochromis* cichlids, which appear to have evolved assortative mating among incipient species in response to sex ratio distortion in hybrid clutches (Seehausen, van Alphen, & Lande, 1999). The mechanisms underlying the lack of adult F1 females are also unknown. Investigation into the genetics of sex determination in darters would add insight into why female hybrids are missing from F1 hybrid clutches.

We also documented substantial postzygotic isolation between orangethroat and rainbow darters in the backcross generation. When wild‐caught F1 males were crossed to females of both parental species, backcross clutches in both directions had dramatically reduced fertilization success, hatching success, and larval survival compared to clutches resulting from purebred parental crosses. The dramatic reduction in fertilization success observed in the backcross clutches is likely attributable to genetic incompatibilities being unmasked in backcross progeny, rather than F1 hybrid male sterility. Wild‐caught F1 hybrid males did not exhibit any morphological or histological defects of the testes, and fertilized eggs in 10 of 12 backcrosses. Many of the backcrosses also produced embryos with obvious developmental abnormalities that died before hatching (R. Moran, pers. obs.). A small number of progeny resulting from backcrosses to both parental species were able to survive until the free feeding larval stage, indicating that although intrinsic postzygotic isolation between orangethroat and rainbow darters is very high, it is not complete. This has implications for the evolution of mating preferences in this system, in which previous studies have shown to be consistent with reinforcement (see below) (Moran & Fuller, 2018; Moran et al., 2017).

### 4.2 Implications for reinforcement

Genomewide sequence data indicated high genetic differentiation and a 1% nucleotide divergence between orangethroat and rainbow darters. Heterozygosity and nucleotide diversity were generally low in both species, but higher in rainbow darters. This observation is consistent with previous analyses of genetic diversity in these species (Moran et al., 2017) and may reflect higher levels of population connectivity in rainbow darters compared to orangethroat darters (Page, 1983).

In particular, our results indicate that F1 hybrids form in nature and that we can accurately diagnose hybrid males based on color pattern attributes that are intermediate between the two purebred parental species. Molecular markers have also been used to document the presence of naturally occurring F1s, F2s, and backcrosses in both directions between rainbow darters and the orangethroat darter clade species *E. bison* (the buffalo darter) (Bossu & Near, 2013), and F1 hybrids between rainbow darters and the orangethroat darter clade species *E. uniporum* (the current darter) (Moran et al., 2017).

The evidence for contemporaneous hybridization between orangethroat and rainbow darters together with the high levels of postzygotic isolation observed provides critical support for previous claims that reinforcement is responsible for driving the patterns of RCD (and potentially ACD) documented in this system (Moran & Fuller, 2018; Moran et al., 2017; Zhou & Fuller, 2016). Orangethroat and rainbow darter males from sympatric populations consistently show strong biases for mating with conspecific females and fighting with conspecific males (when given a choice between orangethroat or rainbow darters). Such biases are not present in orangethroat and rainbow darters that occur in allopatry with respect to one another (Moran & Fuller, 2018). The presence of strong postzygotic isolation and ongoing hybridization between these species has likely created selection favoring the high levels of behavioral isolation observed in sympatry compared to allopatry. Selection to avoid interspecific male–male aggressive interactions in sympatric populations (i.e., ACD) presumably acts to facilitate the co‐occurrence of these species in such close proximity to one another in riffle microhabitats during the spawning season. In turn, the fact that orangethroat and rainbow darters occur syntopically on the same spawning grounds increases the potential for hybridization, which can then further fuel RCD via reinforcement. In this manner, RCD and ACD may act in a positive feedback loop to strengthen male behavioral biases against heterospecific females and males (Moran & Fuller, 2018; Vallin, Rice, Bailey, Husby, & Qvarnström, 2012).

The lack of behavioral biases in wild‐caught F1 males stands in contrast to the strong biases that were previously documented for sympatric male orangethroat and rainbow darters from the same drainage (Moran & Fuller, 2018; Moran et al., 2017). Wild‐caught F1 hybrid males pursued females of both parental species equally and engaged in a comparable amount of aggressive interactions with males of both parental species. In the same way, females and males of both parental species did not show any mating or fighting biases against hybrid males. These observations suggest that F1 males are behaviorally intermediate between the two parental species, similar to the pattern we observed in male color pattern. Furthermore, it has previously been argued that in sympatry, selection favors males who fight with conspecific males (over access to conspecific females) and ignore heterospecific males, in order to avoid costly, unnecessary aggression (Moran & Fuller, 2018; Moran et al., 2017). The fact that F1 males engage in contests with males of both parental species suggests that they may pay the costs associated with increased fighting by engaging males of both species.

Evidence from the present study also supports the hypothesis that cascade reinforcement is responsible for the surprisingly high levels of male‐driven behavioral isolation present between species within the orangethroat clade (Moran & Fuller, 2018). By promoting the evolution of mating traits, reinforcement between two species can incidentally cause behavioral isolation among populations within a single species, termed cascade reinforcement (reviewed in Comeault and Matute 2016). Overtime, cascade reinforcement can cause isolated populations within one species that is experiencing reinforcement with a close relative to diverge to such an extent that they are considered distinct species. We hypothesize that such a phenomenon is occurring in orangethroat darters as a correlated effect of reinforcement with rainbow darters. Males from orangethroat clade species that do not co‐occur with one another but do occur sympatrically with rainbow darters exert strong preferences for conspecific over heterospecific orangethroat darter females (Moran et al., 2017). It is possible that the parallel occurrence of reinforcement selecting for increased behavioral isolation between sympatric rainbow darters and multiple species within the orangethroat clade has incidentally led to mismatches in mating preferences and behavioral isolation between species within the orangethroat clade. The alternative hypothesis that sexual selection within species is responsible for this pattern is unlikely, as populations of orangethroat darters that are allopatric from other species in the orangethroat darter clade and from rainbow darters have no detectable levels of behavioral isolation (Moran & Fuller, 2018).

### 4.3 Conclusions

We used genomic data to demonstrate that hybridization is ongoing between orangethroat and rainbow darters. These species were previously thought to lack substantial postzygotic isolation, but we observed dramatically skewed sex ratios in F1s and a high degree of inviability in backcrosses. The results of this study demonstrate that selection to avoid hybridization may be more important than previously thought in darters. Our findings also inform our understanding of how speciation occurs in a highly diverse vertebrate group with traditional sex roles and dimorphism but no apparent female mate preferences. Darters provide a unique example of how male preferences alone can promote mating and fighting trait evolution concurrently between sympatric and allopatric lineages. The extensive amount of postzygotic isolation present between orangethroat and rainbow darters suggests that reinforcement promotes the previously documented patterns of RCD in male mating preferences between these species (Moran & Fuller, 2018), which may incidentally favor the evolution of ACD in male aggressive biases. Furthermore, this implies that cascade effects of reinforcement may be responsible for the evolution of male‐driven behavioral isolation between recently diverged lineages within the orangethroat darter clade that occur sympatrically with rainbow darters (Moran et al., 2017). Darters provide an intriguing study system for future investigations into the genetics/genomics of hybridization, reinforcement, and speciation.

## CONFLICT OF INTEREST

None declared.

## AUTHOR CONTRIBUTIONS

RLM, MZ, and RCF designed the experiments. RLM drafted the manuscript. RCF provided assistance with revisions. RLM and MZ conducted the experiments. RLM conducted data analyses. JMC advised on data analyses, contributed to the data analysis tools, and commented on the manuscript.

## DATA ACCESSIBILITY

Data from the behavioral assays and color analyses are available at the Dryad Digital Repository: https://doi.org/10.5061/dryad.qf45rf2. The RADseq data have been submitted to the NCBI SRA (accession no. SRP152572).
