# Supplementary material for: Hybridization and postzygotic isolation promote reinforcement of male mating preferences in a diverse group of fishes with traditional sex roles
Source: Ecol Evol. 2018 Aug 24;8(18):9282–94. doi: 10.1002/ece3.4434 (PMC6194240; doi:10.1002/ece3.4434)
Supplement: Supplementary file 1 [file ECE3-8-9282-s001.docx]

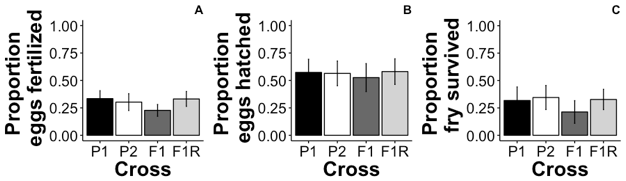


**Figure S1.** Mean proportion (± standard error) of (A) hand stripped eggs that were fertilized (n=10-14 each), (B) fertilized eggs that hatched (n=10-11 each), and (C) hatched fry that survived to 10 months of age (n=5-9 each) in the two parental cross types and two hybrid cross types. P1 = ♀ orangethroat x ♂ orangethroat, P2 = ♀ rainbow x ♂ rainbow, F1 = ♀ orangethroat x ♂ rainbow, F1R = ♀ rainbow x ♂ orangethroat*.*


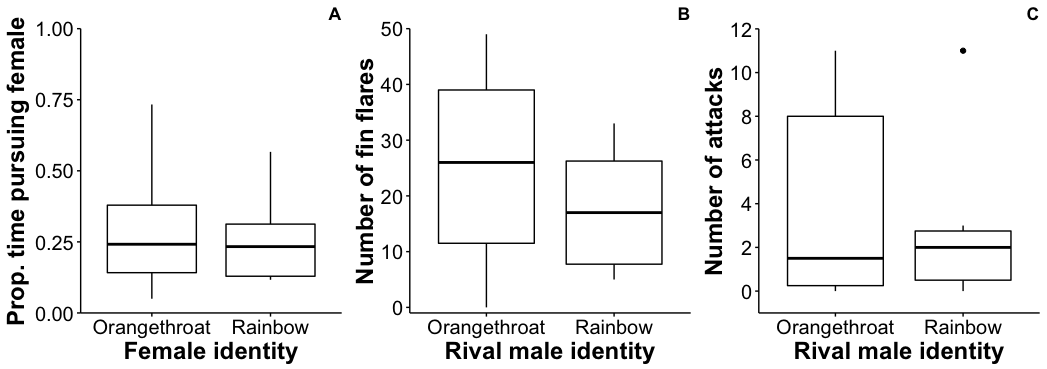


**Figure S2.** Wild-caught orangethroat darter x rainbow darter F1 hybrid male mating and aggressive behavior. (A) Proportion of time hybrid males spent pursuing orangethroat versus rainbow darter females in dichotomous choice trials. (B) Number of fin flares performed by hybrid males towards orangethroat versus rainbow darter rival males. (C) Number of attacks performed by hybrid males towards orangethroat versus rainbow darter rival males.

**
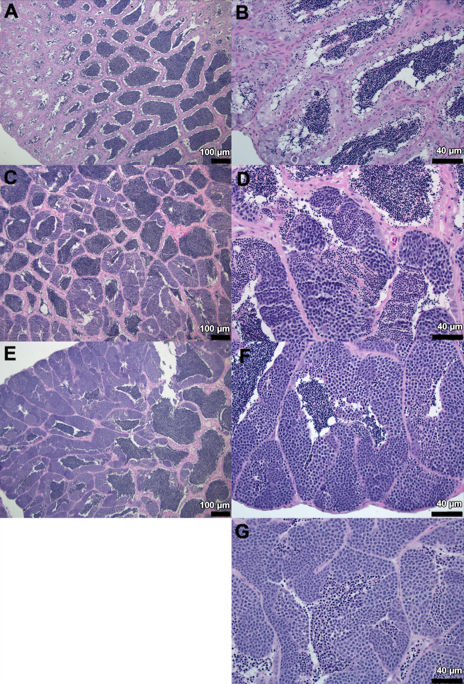
**

**Figure S3.** Cross section of testes from orangethroat darter (A,B), rainbow darter (C,D) and F1 hybrid (E-G) males.


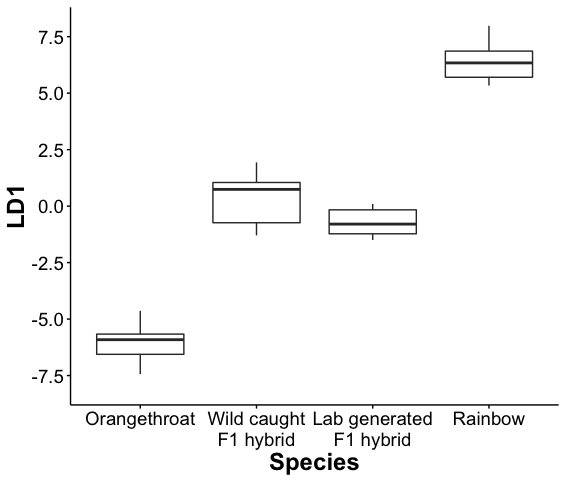


**Figure S4.** Scores for LD 1 of the male color pattern LDA for orangethroat darters, wild-caught F1 hybrids, lab-generated F1 hybrids, and rainbow darters.
