# Supplementary material for: Hybridization and postzygotic isolation promote reinforcement of male mating preferences in a diverse group of fishes with traditional sex roles
Source: Ecol Evol. 2018 Aug 24;8(18):9282–94. doi: 10.1002/ece3.4434 (PMC6194240; doi:10.1002/ece3.4434)
Supplement: Supplementary file 2 [file ECE3-8-9282-s002.docx]

**Table S1.** Collection locations for orangethroat darters, rainbow darters, and F1 hybrids.

| **Species** | **Experiment** | **Latitude** | **Longitude** |
| --- | --- | --- | --- |
| Orangethroat | F1 cross, Backcross | 40.088394 | -88.142504 |
| Rainbow | F1 cross | 40.055556 | -88.091667 |
|  | Backcross | 40.123949 | -88.209260 |
| F1 hybrid | Backcross | 40.116161 | -88.204336 |

**Table S2.** Coefficients of linear discriminants (LDs) from male color pattern LDA in orangethroat darters, rainbow darters, and F1 hybrid males.

| **Measurement type** | **Variable** | **LD1** | **LD2** | **LD3** |
| --- | --- | --- | --- | --- |
| RGB value | Df1R_r | 0.016 | 0.002 | -0.002 |
|  | Df1R_g | 0.044 | -0.009 | 0.014 |
|  | Df1R_b | -0.008 | 0.078 | 0.058 |
|  | Df1B_r | -0.003 | -0.006 | 0.007 |
|  | Df1B_g | -0.003 | 0.011 | -0.015 |
|  | Df1B_b | -0.003 | 0.009 | -0.002 |
|  | Df2R_r | 0.006 | 0.020 | -0.033 |
|  | Df2R_g | -0.004 | 0.008 | -0.026 |
|  | Df2R_b | 0.015 | -0.036 | 0.044 |
|  | Df2B_r | 0.009 | -0.011 | 0.007 |
|  | Df2B_g | -0.003 | -0.001 | 0.019 |
|  | Df2B_b | 0.040 | -0.018 | 0.007 |
|  | AfR_r | 0.007 | -0.005 | -0.001 |
|  | AfR_g | 0.010 | -0.004 | 0.001 |
|  | AfR_b | -0.003 | -0.001 | 0.003 |
|  | AfB_r | -0.030 | 0.009 | 0.025 |
|  | AfB_g | -0.010 | -0.001 | 0.005 |
|  | AfB_b | 0.003 | -0.007 | 0.003 |
|  | LatR_r | -0.002 | 0.005 | -0.021 |
|  | LatR_g | 0.012 | 0.000 | -0.006 |
|  | LatR_b | 0.001 | -0.029 | 0.078 |
|  | LatB_r | 0.012 | -0.014 | 0.000 |
|  | LatB_g | 0.013 | 0.013 | -0.007 |
|  | LatB_b | -0.015 | 0.005 | -0.010 |
|  | CaudR_r | 0.005 | -0.006 | 0.005 |
|  | CaudR_g | -0.003 | 0.002 | 0.005 |
|  | CaudR_b | 0.032 | -0.008 | 0.003 |
| Color proportion | Df1_PB | 1.100 | 1.248 | -1.181 |
|  | Df1_PR | 3.956 | -6.657 | 3.330 |
|  | Df2_PB | 7.123 | -0.927 | 2.941 |
|  | Df2_PR | -1.403 | -4.787 | -0.119 |
|  | Af_PB | 2.121 | -1.079 | 1.291 |
|  | Af_PR | 4.877 | 10.087 | -5.367 |
|  | Body_PB | 5.086 | 2.154 | -2.949 |
|  | Body_PR | -5.217 | 3.768 | 5.815 |
|  | Caud_PR | 7.544 | 3.553 | 3.011 |

Df = dorsal fin, Af = anal fin, Lat = lateral side, Caud = caudal fin, R = red, B = blue, PR = proportion red, PB = proportion blue.

**Table S3**. Results of Structure (Pritchard et al. 2000) analysis on 1,073 SNPs obtained from the 42 individuals used in the backcross experiment (orangethroat darters: n = 18, rainbow darters: n = 18, F1 hybrids: n = 6).

| **K** | **Reps** | **Mean LnP(K)** | **Stdev LnP(K)** | **Ln'(K)** | **\|Ln''(K)\|** | **Delta K** |
| --- | --- | --- | --- | --- | --- | --- |
| 1 | 20 | -34,929.50 | 0.00 | NA | NA | NA |
| 2 | 20 | -11,038.83 | 0.83 | 23,890.68 | 2,5047.61 | 3,0191.22 |
| 3 | 20 | -12,195.76 | 3,987.59 | -1,156.94 | 611.51 | 0.15 |
| 4 | 20 | -12,741.19 | 3,376.52 | -545.43 | 2,060.81 | 0.61 |
| 5 | 20 | -11,225.81 | 524.95 | 1,515.38 | NA | NA |

Summary statistics presented for each value of K were generated using Structure Harvester (Earl and vonHoldt 2012). The optimal value of K (i.e., the number of distinct genetic clusters) was inferred to be 2 using the Evanno method (Evanno et al. 2005), which identifies K as the largest value of Delta K.

**Table S4.** Hybrid indices (h) for each of the six wild-caught F1 hybrid males. The value for h represents the maximum likelihood estimate of the proportion of orangethroat darter (versus rainbow darter) alleles present in each hybrid male. Also shown are the likelihood value and the upper and lower limits of the 95% confidence interval for each individual.

| **Individual** | **h** | **ln(likelihood)** | **Low** | **Up** |
| --- | --- | --- | --- | --- |
| 1 | 0.501 | -766.435 | 0.475 | 0.527 |
| 2 | 0.522 | -307.065 | 0.473 | 0.571 |
| 3 | 0.514 | -711.405 | 0.485 | 0.544 |
| 4 | 0.510 | -764.757 | 0.481 | 0.540 |
| 5 | 0.566 | -324.111 | 0.519 | 0.613 |
| 6 | 0.538 | -823.007 | 0.511 | 0.565 |
