# Supplementary material for: Hybridization and postzygotic isolation promote reinforcement of male mating preferences in a diverse group of fishes with traditional sex roles
Source: Ecol Evol. 2018 Aug 24;8(18):9282–94. doi: 10.1002/ece3.4434 (PMC6194240; doi:10.1002/ece3.4434)
Supplement: Supplementary file 3 [file ECE3-8-9282-s003.docx]

**Supplemental Methods**

*Details of male color pattern measurements*

We photographed laboratory-raised F1 hybrid males once they reached 22 months of age, at which point they exhibited male nuptial coloration. We also photographed the wild-caught putative F1 hybrid males and wild-caught *E. spectabile* and *E. caeruleum* males and females that were used in the backcross and behavioral experiments within 24 hours of their collection. Fish were lightly anesthetized using MS-222 prior to photographing, and then placed on their sides in a petri dish that was filled with water and on a white background. Photographs were taken under fluorescent lighting using a Nikon Coolpix D3300 digital camera with the factory setting for photography in fluorescent lighting. An X-rite ColorChecker Mini Chart (Grand Rapids, MI) was included in each photo to allow for color correction and standardization across photographs. After photographing, fish were transferred to a bucket of aerated water to recover. Raw files of the digital photographs were color corrected and standardized using the inCamera 4.5 plug-in for Adobe Photoshop CC.

We used two categories of color measurements to quantify male color pattern: RGB values of the red and blue coloration on the body and fins, and proportion of red and blue coloration on the body and fins. We used the Color Sampler Tool in Adobe Photoshop CC to measure the RGB value in both the red and the blue areas (if present) on the lateral side of the body, the first dorsal fin, the second dorsal fin, the anal fin. We also measured the RGB value in the red areas on the caudal fin if present. This resulted in a total of 9 separate locations measured on each fish. Each RGB measurement gives a separate value for R, G, and B that range from 0-255 (where a 0, 0, 0 represents pure black and 255, 255, 255 represents pure white). Thus, three values were recorded for each of the 9 color locations, resulting in 27 RGB variables per fish. If red or blue coloration was absent on a given part of a fish (i.e., no anal fin red coloration), the R, G, and B values for that body part-color combination were recoded as not available. The Color Sampler Tool was set to measure 3x3 pixel samples, and each RGB color sample was taken three times at the same location on the same photograph for each fish. The average of these values was then used in color analyses (Zhou et al. 2014; Moran et al. 2017a).

We used ImageJ (version 1.50c4) to measure the proportion of red and blue coloration present on each males’ lateral side of the body, first dorsal fin, second dorsal fin, and anal fin. We also measured the proportion of red coloration present on the caudal fin. This resulted in 9 separate color proportion measurements for each fish. ImageJ’s Threshold Colour plug-in was set to *L*a*b** color space and used to adjust the color threshold of the photographs (Zhou et al. 2014; Moran et al. 2017a). We used *L*a*b** rather than RGB color space, as it allowed for better isolation of the red and blue components of male color pattern. We first used the Polygon Selections tool to trace an outline around the perimeter of the body and each fin one at a time. The Histogram tool was used to obtain a count of the total number of pixels in each of the outlined areas. To isolate only the blue coloration on the fins and body, the Threshold Colour plug-in was set to stop (i.e., exclude) pixels with *L** values above 200 and *b** values above 140. We then converted the image to binary, causing all of the isolated blue pixels to be changed to black. We then used the Polygon Selections tool to trace around each of the fins and the body, and used the Histogram tool to produce a count of the total number of black pixels in each region; this number was divided by the original total number of pixels counted in each region (before the blue coloration was isolated) to obtain the proportion of blue coloration on each fin and the body. The same process was followed to isolate and measure red coloration, but the Threshold Colour plug-in was set to stop pixels with *L** values above 200 and *a** values below 130. If a color was absent on a given part of a fish, the color proportion was recorded as 0.
